# 

**Published:** 1898-11

**Authors:** 


### THE GEORGIA MEDICAL ASSOCIATION.

It is perhaps a little early to begin talking about the next meeting of our State Medical Association, but we do this in order, if possible, to arouse early enthusiasm. The next meeting will be held in Macon, Ga., and it will be the celebration of its fiftieth anniversary. Macon was very rightly chosen as the place of meeting, since it was in the Central City, fifty years ago, that the Association was founded. We expect to have a great celebration on that occasion, and we urge now upon every member to make his preparations preparatory to being present. Socially, besides many other ways, Macon is one of the most delightful places in the South, and there has never been a meeting of the Association held in that city but what all present wished to return again. Socially, then, we are assured of the success of the meeting. But what we want to do is to have a program of medical papers which will be worthy of the occasion and of the physicians who represent the old State of Georgia. Last year the meeting of our Association was not up to its usual standard, and this was largely due to the fact that the place of meeting, Cumberland Island, was too far away for the majority of physicians. Macon, however, is unusually central, and the full quota of membership should be present.

---

That distinguished Southern surgeon and teacher, Dr. Floyd W. McRae, of the Atlanta College of Physicians and Surgeons, has been placed in an awkward position by the American Medical Association. First, he was elected by the Association to deliver the annual address in surgery at the Columbus meeting next year. Next, he was declared not eligible to membership unless he resign his professorship in the college. For the Association, without dissenting vote, passed the resolution declaring that no physician can hereafter be a delegate in that body who is a member of any faculty, or a graduate of any college which does not require a four years' graded course of instruction; and the announcement of the college in which Dr. McRae teaches is outspoken for only three years! This eminent Southern teacher is not the only member of long standing who will be affected—fully 40 per cent. of the permanent members of the American Medical Association are graduates of schools which will next year (for the last time) grant the degree of "doctor of medicine" to men who have attended only three annual courses of instruction. The meeting at Columbus next year will be a miserable failure if the resolution adopted at Denver be enforced, since the most enthusiastic workers of that body are from the South and West, where fully nine-tenths of the doctors are graduates of "three-year schools." If the resolution had read "after 1900" instead of "hereafter," there would have been no trouble, as all the reputable schools of the country are to require the "four-year course" after the next session.—*American Journal Surgery and Gynecology.*

---

THE *Medical Age*, in its Items and Notes, has this to say: "The doctors of Sweden never send bills to their patients. If you have occasion to call a physician you will find him not only skilled in his profession, but a highly educated and honorable gentleman. You will also have a proof of the honesty of the Swedes and their friendly confidence in each other. What you shall pay is left entirely to your own choice. The rich may pay him liberally, whether they have need of his services or not, if he has only been retained by them. The poor may pay him a small sum and the very poor pay him nothing. Yet he visits the poor as faithfully as he does the rich. A similar custom prevailed up to the middle of the present century in some of the most remote portions of the Highlands of Scotland. There the doctor collected his entire year's bills on a certain market day in summer, getting perhaps five or ten pounds from the larger farmers, but only as many shillings from the poorer crofters."

What an ideal life must be that of the Swedish physician! Think of such a condition of affairs existing here in America! We fear that such a degree of honesty could never be acclimated in this land of ours.

---

IT seems as if the H. K. Mulford Co., of Philadelphia, have been appropriating a good deal which did not belong to them, if we may judge from the *Therapeutic Notes* issued by the well-known firm of Parke, Davis & Co., of Detroit. According to the statement of the Philadelphia firm, the antitoxin manufactured by them is that which has been adopted by the California State Board of Health as being the best product made, and therefore to be used. Now comes the Parke-Davis Co., who, under sworn statements from the ex-president of the California Board of Health and the present secretary, state that the Mulford Co. have inserted their own name in the place of Parke, Davis & Co. in the resolution which was really passed by the Board of Health.

These published affidavits certainly place the Mulford Co. in an unenviable light, to say the least, and if what they have been guilty of is true—and it certainly looks that way—then such action should be condemned *in toto* by the whole profession.

### REPRINTS RECEIVED.

Endemic Leprosy in Louisiana. By Isadore Dyer, Ph.B. (Yale), M.D., New Orleans.

A Rare Form of Bone Atrophy following an Ununited Fracture as seen by the X-ray. By Eugene R. Corson, M.D., Savannah, Ga.

The Adirondacks in Winter for Tubercular Patients. By Sargent F. Snow, M.D., Buffalo, N. Y.

Acute Chloral Dementia Simulating Paretic Dementia. By Henry W. Coe, M.D., Portland, Oregon.

A Contribution to the Surgery of Gastroptosis and Enteroptosis: Preparation of the Patient for Operation. By Byron B. Davis, M.D., Omaha, Neb.

The Advantages of Physical Education as a Prevention of Disease. By Charles Denison, M.D., Denver, Col.

A Clinical Record of Two Cases of Meniere's Disease, with Remarks Thereon; Eighteen Years of Personal Observation of Tuberculosis in Asheville, N. C. By John Hey Williams, A.M., M.D., Asheville, N. C.

The Aseptic Animal Suture; Its Place in Surgery. By Henry O. Marcy, M.D., A.M., LL.D., Boston.

